# Oral swabs as a proxy for direct ruminal microbiome sampling in Holstein dairy cows is correlated with sample color

**DOI:** 10.3389/fmicb.2024.1466375

**Published:** 2024-09-17

**Authors:** Joseph H. Skarlupka, Madison S. Cox, Andrew J. Steinberger, Dino L. Sbardellati, Jennifer C. McClure, Derek M. Bickhart, Andrew J. Scheftgen, Ibrahim Zuniga-Chaves, Luke A. Wolfe, Eric Paget, Charles Skadron, Nithya Attipetty, Garret Suen

**Affiliations:** ^1^Microbiology Doctoral Training Program, University of Wisconsin–Madison, Madison, WI, United States; ^2^Department of Bacteriology, University of Wisconsin–Madison, Madison, WI, United States; ^3^Department of Allergy and Infectious Disease, University of Washington School of Medicine, Seattle, WA, United States; ^4^Microbiology Graduate Group, University of California, Davis, Davis, CA, United States; ^5^USDA Dairy Forage Research Center, Madison, WI, United States; ^6^Hendrix Genetics, Boxmeer, Netherlands

**Keywords:** buccal swab, oral community, rumen microbiota, swab color, bacteria, fungi

## Abstract

Using oral swabs to collect the remnants of stomach content regurgitation during rumination in dairy cows can replicate up to 70% of the ruminal bacterial community, offering potential for broad-scale population-based studies on the rumen microbiome. The swabs collected from dairy cows often vary widely with respect to sample quality, likely due to several factors such as time of sample collection and cow rumination behavior, which may limit the ability of a given swab to accurately represent the ruminal microbiome. One such factor is the color of the swab, which can vary significantly across different cows. Here, we hypothesize that darker-colored swabs contain more rumen contents, thereby better representing the ruminal bacterial community than lighter-colored swabs. To address this, we collected oral swabs from 402 dairy cows and rumen samples from 13 cannulated cows on a research farm in Wisconsin, United States and subjected them to 16S rRNA sequencing. In addition, given that little is known about the ability of oral swabs to recapitulate the ruminal fungal community, we also conducted ITS sequencing of these samples. To correlate swab color to the microbiota we developed and utilized a novel imaging approach to colorimetrically quantify each swab from a range of light to dark. We found that swabs with increasing darkness scores were significantly associated with increased bacterial alpha diversity (*p* < 0.05). Lighter swabs exhibited greater variation in their community structure, with many identified amplicon sequence variants (ASVs) categorized as belonging to known bovine oral and environmental taxa. Our analysis of the fungal microbiome found that swabs with increasing darkness scores were associated with decreased alpha diversity (*p* < 0.05) and were also significantly associated with the ruminal solids fungal community, but not with the ruminal liquid community. Our study refines the utility of oral swabs as a useful proxy for capturing the ruminal microbiome and demonstrates that swab color is an important factor to consider when using this approach for documenting both the bacterial and fungal communities.

## 1 Introduction

Dairy cows are reliant upon their rumen and the microbes found therein to provide nutrients from their otherwise indigestible feed (Flint et al., 2008; Van Soest, 1994). The rumen plays an important role in driving milk production efficiency (MPE) and the production of greenhouse gasses such as methane (Jewell et al., 2015; Cox et al., 2021; Hegarty et al., 2007). The rumen can also disrupt cow productivity due to dysbiosis where the ruminal microbiome is disturbed leading to metabolic disorders and other conditions such as subacute ruminal acidosis (SARA)(Stone, 2004; Kleen et al., 2003). Characterization of the rumen microbiome is of great interest to researchers and producers due to its central role in health and productivity.

Several studies suggest large-scale sampling of the ruminal microbiome across a herd would allow for advances in dairy cow productivity (Tapio et al., 2016; Kittelmann et al., 2015). This includes selection of a specific microbiome through genetic breeding efforts to increase MPE, decrease methane production, or to aid the in early detection of conditions like SARA. Previous studies have shown the inclusion of ruminal microbial data improves models predicting feed efficiency metrics like milk production and dry matter intake (Marcos et al., 2024; Martinez Boggio et al., 2024). These studies benefit from large sample sizes, but a complicating factor in documenting the ruminal microbiome lies in our inability to sample this community on a large scale. Unlike other mammalian systems, proxies such as fecal material do not recapitulate the ruminal microbiome (Tapio et al., 2016). Other methods to access the ruminal microbiome, such as stomach tubing or rumenocentesis, are invasive, laborious, and collect mainly ruminal liquids, which has a microbial community distinct from the rumen solids (Jewell et al., 2015). Collection of samples via stomach tubing often requires special restraints, like a chute system, and require multiple people to collect a sample from a single animal. Moreover, the current gold standard—sampling via a cannula—is impossible to deploy at a large scale on commercial dairies due in part to the costly surgeries to install the cannula and the time required to sample individual animals (Duffield et al., 2004; Skarlupka et al., 2019).

In recent years, using oral contents as a proxy for sampling the ruminal microbiome has garnered significant interest (Tapio et al., 2016; Kittelmann et al., 2015; Marcos et al., 2024; Amin et al., 2021; Miura et al., 2022; Mott et al., 2022; Edwards et al., 2023), as they offer multiple advantages over other sampling options. First, no extra restraints are required. Cows can be held in either head-lock or free-tie stalls that are common to dairy operations. Second, oral swabbing is also less invasive, can be rapidly collected (<1 min), and only requires one person for sample collection, allowing for many animals to be quickly sampled. Previous work by our group, and others, have demonstrated the potential for oral swabbing as an effective proxy, with the ability of swabs to recapitulate as much as 70% of the ruminal bacterial community (Tapio et al., 2016; Kittelmann et al., 2015; Young et al., 2020). By capturing the remnants of feed regurgitated into the mouth during rumination, oral swabs have the advantage of being noninvasive, and, given its easy and rapid application at the herd level, are amenable to “convenience” sampling. However, a significant challenge in deploying oral swabs at a commercial level lies in the significant variation observed across swab samples from different times or conditions. For example, we previously showed that obtaining ~70% congruence between the microbiomes obtained from oral swabs and their corresponding cannula-collected ruminal samples required sampling at specific times under controlled conditions (Young et al., 2020). This is likely not feasible on a commercial dairy, where an ideal application would be “convenience” sampling that does not disrupt the day-to-day management operations of producers.

One key variance in oral swab quality is the color of the swab, which, in animals fed with a total mixed ration (TMR), ranges from light to dark with varying shades of brown. We hypothesize that swab color may be determined by how much ruminal contents have been collected. If a swab is collected during or directly after rumination, it is likely there will be more rumen content in the mouth available for collection by swabbing. A lighter colored swab is thus likely a function of the elapsed time between rumination and the time of oral swab collection. As such, it is likely that darker colored swabs are more reflective of the ruminal microbiome and thus swab color may be an effective indicator of a swab to accurately capture the rumen microbiome.

To address this, we collected oral swabs from all lactating animals on a large research dairy farm in south-central Wisconsin, United States. We developed and applied a novel method of colorimetrically quantifying swab color and assigning an objective color score to each swab. Next-generation sequencing was used to characterize both the bacterial and fungal communities captured by the swabs. We then sought to determine if the color of the swab is directly correlated to the structure of a swab’s bacterial and fungal communities, with darker swabs exhibiting a more similar microbial composition to their associated ruminal microbiomes.

## 2 Methods

### 2.1 Animal use

Experimental protocols were approved by the Institutional Animal Care and Use Committee at the University of Wisconsin–Madison (Protocol ID: A005902).

### 2.2 Sample and metadata collection

Samples were collected as part of a larger project at a University of Wisconsin-Madison research farm in Arlington, WI in October 2019. Swab samples from 402 animals were collected 1–2 h prior to morning feeding. To minimize the introduction of environmental microbes and encourage rumination, farm staff removed feed from the night before an hour before sample collection. Hay was used sparingly to entice animals into headlocks. Sterile flocked swabs (Puritan PurFlock Ultra; Puritan Medical Products Co LLC; Maine, United States) were used in pairs to scrape the cheek at the back of the mouth of lactating cows for 15 s. Swabs were placed into individual 2 mL screw-cap tubes with 0.5 mL of chilled 1x phosphate-buffered saline (PBS) before placement on ice. Swabs were then returned to the lab and stored at −20°C before further analysis.

Of the 402 animals, 13 were cannulated. Whenever we collected a swab sample from a cannulated animal, rumen samples were also collected at the same time via direct sampling through cannula. Rumen contents were squeezed through four layers of sterile cheesecloth to separate the liquid and solid phases. Liquid and solid contents were placed into separate 50 mL conical tubes and stored on ice until returned to the lab and stored at −20°C before further analysis.

**Figure 1:**
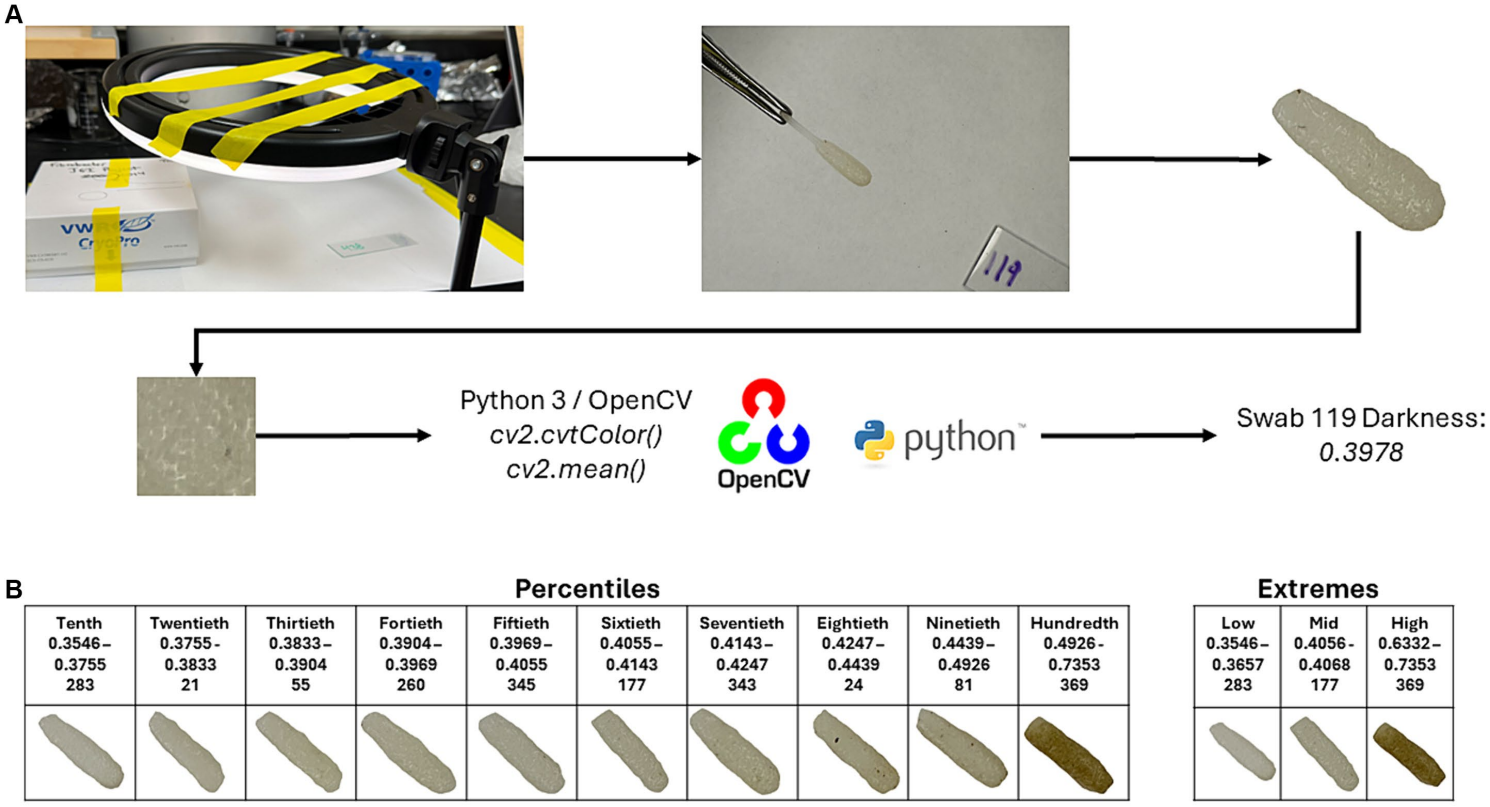
Workflow for swab darkness calculations. **(A)** Swabs were photographed under a ring light acting as the only light source. The swab head was edited out, and a 100 × 100 pixel square was taken from the center point of the swab and converted to grayscale using the OpenCV package. The mean darkness was then calculated. The cutoffs for the Percentiles and Extremes sets, swab ID number, and representative images are displayed in **(B)**.

To determine the darkness of the swabs, images were taken of the individual swabs, and the swab was trimmed out of each image using Adobe Photoshop (v25.6.0; Adobe Inc., San Jose, CA). Photos were taken using a light ring (Weilisi; 10″ Ring Light) in a room with no source of outdoor lighting. Because DNA extractions of the samples occurred before the photographing of the swabs, the swab’s pair (which was collected at the same time as the original) was used for imaging. The CV2 package (v4.9.0.80) in Python (v3.12.3) was used to crop a 100 × 100 pixel square at the center of the swab. The cropped image was then converted to grayscale using the *cv2.cvtColor()* function. After converting to grayscale, an average pixel darkness was calculated, with the given value being used for later statistical analyses (Figure 1A). Code for the cropping, grayscale conversion, and average pixel darkness is available at https://github.com/JSkar/SwabColor.

### 2.3 DNA extraction, PCR amplification, and sequencing

For swabs, prior to extraction, 0.5 mL of 1x PBS buffer was added to the tube containing the swab (for a total of 1 mL of buffer). Tubes were placed into a bead beater for 2 min to dislodge cells from the swabs. The liquid was removed and centrifuged at 15,000 × g for 15 min to pellet the cells. Enough supernatant was removed to leave 150 uL of liquid, where the pellet was then resuspended. For rumen liquids, samples were spun in a centrifuge at 4,000 × g for 1 h. The supernatant was removed, and the pellet was resuspended with 5 mL of PBS before storage at −80°C. For rumen solids, samples were prepared for extraction by using a stomacher (Seward Stomacher 400; Seward Inc., West Sussex, UK), which uses two paddles to agitate the sample and remove fiber-adherent cells from the solids and further break them into smaller pieces. A total of 10 mL by volume of rumen solids were mixed with 45 mL of 1x PBS in a stomacher bag with a 0.5 mm filter insert (BA6040/CLR/STR; Seward Inc.). After 5 min, the corner of the bag was cut, and the liquid was removed. The buffer was then spun in a centrifuge at 4,000 × g for 1 h to concentrate the cells. The supernatant was removed, and the pellet was resuspended with 5 mL of PBS before storage at −80°C until further use.

A Zymogen Quick-DNA HMW MagBead Kit (D6060; Zymo Research Corp., Irvine, CA, United States) adapted for a 96-well plate format was used for DNA extraction. In short, cells were lysed using a lysozyme + heat digestion at 37°C for 30 min followed by an SDS + proteinase K + heat digestion at 55°C for 20 min. DNA was isolated and purified with magnetic beads and wash buffer before being eluted using 1x Tris-EDTA buffer. Purified DNA was stored at −20°C until further use.

DNA was quantified using Qubit fluorometer reagents (Invitrogen; Waltham, MA, United States) and a Synergy 2 microplate reader (BioTek; Winooski, VT, United States). Sequencing of the bacterial community followed a protocol outlined previously (Young et al., 2020). Briefly, the V4 region of the 16S rRNA gene was amplified using barcoded universal primers with adapters suitable for sequencing on an Illumina MiSeq (Kozich et al., 2013) (V4 Region Primers: F-GTGCCAGCMGCCGCGGTAA, R-GGACTACHVGGGTWTCTAAT; Illumina Adapters: F-AATGATACGGCGACCACCGAGATCTACAC, R-CAAGCAGAAGACGGCATACGAGAT). A 25uL polymerase chain reaction (PCR) containing 12.5uL of 2x KAPA HiFi HotStart ReadyMix (KAPA Biosystems; Wilmington, MA, United States), 1–15 ng of DNA, 0.2umol/L each of forward and reverse primers, and ultra-pure water was performed on a Bio-Rad S1000 thermocycler (Bio-Rad Laboratories, Hercules, CA) using the following program: 95°C for 3:00, 30 cycles of 95°C for 3:00, 55°C for 0:30, 72°C for 0:30, ending with a final extension at 72°C for 5:00. Full primer sequences can be found in Supplementary Table S1.

Fungal characterization was performed by amplifying the ITS-2 region of the fungal nuclear ribosomal ITS gene (ITS-2 Region Primers: F-AACTTTYRRCAAYGGATCWCT, R-AGCCTCCGCTTATTGATATGCTTAART) (Taylor et al., 2016). Although the rumen fungal community is highly specific and is dominated by the *Neocallimastigaceae* family, the presence of oral and environmental fungi in the swabs necessitated the use of a more generalized fungal primer. The primers contained custom barcodes and Illumina-specific adapters, as detailed in Kozich et al. (2013). A 25uL PCR reaction containing 12.5uL of 2x KAPA HiFi HotStart ReadyMix (KAPA Biosystems; Wilmington, MA, United States), 1–15 ng of DNA, 0.2umol/L each of forward and reverse primers, and ultra-pure water was performed on a thermocycler using the following program: 95°C for 3:00, 30 cycles of 95°C for 3:00, 58°C for 0:30, 72°C for 0:30, ending with a final extension at 72°C for 5:00.

Amplification was confirmed on a 1% low melt agarose gel with SYBRSafe (Invitrogen; Waltham, MA, United States) and DNA was purified from gel-excised PCR products. Specifically, bands at ~380 base pairs (bacterial) or 250–600 base pairs (fungal) indicated successful amplification, and these regions were excised, and DNA was purified using ta Zymoclean Gel DNA Recovery Kit (Zymo Research Corp.; Irvine, CA, United States). Purified PCR products were quantified using Qubit fluorometer reagents on a microplate reader. Bacterial samples were pooled equimolarly to 4 nM and sequenced on an Illumina MiSeq using a v2 2 × 250 bp sequencing kit (Illumina; San Diego, CA, United States) at 11 pmol/L with 10% PhiX control, whereas pooled fungal libraries were sequenced on an Illumina MiSeq using a v3 2 × 300 kits (Illumina; San Diego, CA, United States) at 11 pmol/L with 10% PhiX control. Raw reads for this study are publicly available in the NCBI’s Short Read Archive under BioProject number PRJNA1117920.

### 2.4 Sequence cleanup

The resulting bacterial sequencing files were cleaned using DADA2 (v1.24.0) in R (v4.2.1) (R Core Team, 2021; Callahan et al., 2016). DADA2 takes an input of paired end reads and generates an ASV table and taxonomy assignments for the combined reads. The DADA2 pipeline for sample processing was used with the following parameters for the filter and trimming steps: *truncLen = c(230,200), maxN = 0, maxEE = c* (Jewell et al., 2015; Hegarty et al., 2007)*, truncQ = 2*. Chimeras were removed using the *removeBimeraDenovo()* command. Taxonomy was assigned to each ASV at the species level using the reference strains found in the SILVA reference database (v138.1) (Quast et al., 2012) and the *phangorn* package (v2.11.1) was used to generate a phylogenetic tree (Schliep, 2011).

The fungal sequencing files were cleaned using DADA2 in R (R Core Team, 2021; Callahan et al., 2016). The DADA2 ITS pipeline for fungal sample processing was used by first trimming primers using cutadapt (v3.5), and filtering sequences with ambiguous bases (Martin, 2011). In cutadapt, the forward and reverse primer sequences used as inputs were AACTTTYRRCAAYGGATCWCT and AGCCTCCGCTTATTGATATGCTTAART, respectively. The sequences were filtered and trimmed using the *filterAndTrim* command and the following parameters: *truncLen = c(270,250), maxN = 0, maxEE = c* (Jewell et al., 2015; Hegarty et al., 2007)*, truncQ = 2*. Chimeras were removed using the *removeBimeraDenovo()* command. Taxonomy was assigned using the UNITE ITS database (Version 9.0, Release 2023-07-18) and the *phangorn* package (v2.11.1) was used to generate a phylogenetic tree (Schliep, 2011).

Bacterial and fungal ASV tables, taxonomic identifications, phylogenetic trees, and metadata were then combined into separate phyloseq objects using the R package *phyloseq* (v1.42.0) (McMurdie and Holmes, 2013). Within *phyloseq*, mitochondrial, chloroplast, and archaeal sequences were removed from the bacterial dataset (*subset_taxa(…, Order! = “Chloroplast”); subset_taxa(…, Family! = “Mitochondria”); subset_taxa(…, Kingdom! = “Archaea”)*). For statistical analyses that required a rarefied dataset, samples were rarefied (7,000 for bacteria, 2,800 for fungi). Following rarefaction of the bacterial samples, 364 swabs, 13 rumen liquid, and 12 rumen solid samples remained. Rarefaction of the fungal samples resulted in 376 swabs, 12 rumen solid, and 7 rumen liquid samples remaining. After rarefaction, we used an abundance cutoff where any ASV for which a given sample did not contain at least 10 sequences were removed.

### 2.5 Statistical analyses

Following cleanup and normalization, darkness scores were used to split swabs into sets using two different criteria from which comparisons were made: (1) All samples split into 10 groups by decile percentiles of darkness scores with group sizes ranging between 33–39 (Bacterial) or 36–40 (Fungal) (Percentiles Groups), and (2) 3 groups split into extremes (Extremes Group). Group sample sizes of the Extremes Group were determined by the number of rumen samples to maintain even sample numbers between the swab groups, rumen solids, and rumen liquids. To form the Extremes Groups, the highest and lowest 13 samples by darkness scores were placed into “High” and “Low” groups, respectively, with a third “Mid” group representing the 13 samples that fell within the middle of the range of darkness scores.

Alpha diversity values were calculated using the *phyloseq* and *vegan* (v2.6–4) packages (*estimate_richness(…, measures = c(“Chao1,” “Shannon,” “InvSimpson”))*) in R (R Core Team, 2021; McMurdie and Holmes, 2013; Oksanen et al., 2022). To test differences in alpha diversities between groups the *stats* package in R was used (v4.2.1) (R Core Team, 2021). Normality was determined using *Shapiro.test()*. The Kruskal-Wallis test was used for our non-normal datasets, with pairwise comparisons conducted for significant results for both the Percentiles and Extremes groups using the Wilcoxon Rank Sum test and correcting for multiple comparisons (*Kruskal.test()*; *pairwise.wilcox.test(…, p.adjust.method = “fdr”)*). To test correlations between darkness scores and alpha diversity scores, Spearman’s rank correlation was used (*cor.test(…, method = “spearman”)*).

To visualize the overall structure of the communities, samples were merged based on their assignment in the Percentiles and Extremes groups. Average relative abundance was calculated and the 10 most abundant phyla were plotted for the Percentiles and Extremes groups using the *plot_bar()* function in *phyloseq*. Beta diversity was calculated in the *phyloseq* package using the *distance()* function, followed by the *ordinate()* function to generate Principal Coordinates Analysis (PCoA) plots. A PERMANOVA test was used to test swab color groups vs. rumen solid vs. rumen liquid (*adonis2()*). Plots were generated for both the Extremes and Percentiles groups. To determine if the darkness of the swab is associated with the swab’s community structure, the axis locations were extracted from the plot and a linear model was fit using the darkness scores and the two axes using the *lm()* function. To further determine the relationship between swab darkness score and their relatedness to the rumen liquid or solid communities, the average distance of each swab to the rumen solid or rumen liquid coordinates were calculated and pairwise comparisons were determined between groups within the Extremes and Percentiles sets; Spearman’s rank correlation was used to determine correlations between the darkness scores and distances.

To determine ASVs significantly different in abundances between groups, we first used the *simper()* function to determine the average contribution of ASVs to the dissimilarity between groups of interest. For ASVs with a SIMPER score > 1%, the *Kruskal.test()* function was used to test for significant differences in abundances after FDR correction. To visualize these results, a heatmap was generated displaying the relative abundance of ASVs that were significantly different between either the High/Low groups (Extremes Set) or the Tenth/Hundredth decile groups (Percentiles Set).

Highly prevalent ASVs in the bacterial and fungal communities were identified for the rumen solid and liquid fractions. Bacterial ASVs with >80% prevalence in either fraction were considered highly prevalent. Due to the slightly lower sequencing quality present in the fungal samples, we considered any fungal ASV with >50% prevalence in either fraction to be highly prevalent. We then determined which prevalent ASVs were present in our swab samples and tested the relationship between the darkness scores and the percentage of the highly prevalent ASVs captured by the swabs.

## 3 Results

### 3.1 Sampling

Sampling took place on the week of October 14, 2019. In total, 402 oral swabs were collected in duplicate. Of those 402 animals, 13 were cannulated, and rumen solid and liquid samples were collected from those animals. One rumen solid sample was lost before sample processing (Swab *n* = 402, Liquid *n* = 13, Solid *n* = 12).

### 3.2 Swab photographing results

To quantify each oral swab according to color, we developed a system whereby an image of each swab was obtained in a consistent and controlled environment. Each image was then processed, and the swab was assigned a colorimetric pixel score that ranged from 0 (white) to 1 (black). After image processing, swab color scores ranged between 0.3546 and 0.7387 with a median of 0.4053. To best understand the influence of swab color on the ability of each swab to recapitulate the ruminal microbiome, we divided our swabs into two sets: (Flint et al., 2008) an “Extremes” group which considered only the top and bottom 13 samples according to pixel color and (Van Soest, 1994) a “Percentiles” group, which divided the swabs into deciles (10 equal percentile groups). Score cutoffs for the different groups in both sets, along with a representative photo of that group, can be found in Figure 1B. We did not have a paired swab for image sampling for 6 samples, bringing the total number of swab samples with darkness scores to 396.

### 3.3 Bacteria

#### 3.3.1 Sequencing and cleanup

A total of 427 samples were sequenced resulting in a total of 14,403,141 reads, with an average of 33,731 reads per sample (±42,251) and ranging from 5,059–352,630 reads. After filtering, 11,988,295 reads remained, with an average of 28,075 reads per sample (±35,208). The number of reads per sample ranged from 4,179 to 295,963. After cleanup and normalization, there were a total of 364 swabs, 12 pairs of rumen samples, and one rumen liquid sample for which there was not a paired solid (Liquid *n* = 13; Solid *n* = 12).

#### 3.3.2 Correlations between the darkness of swabs and the bacterial community

**Figure 2:**
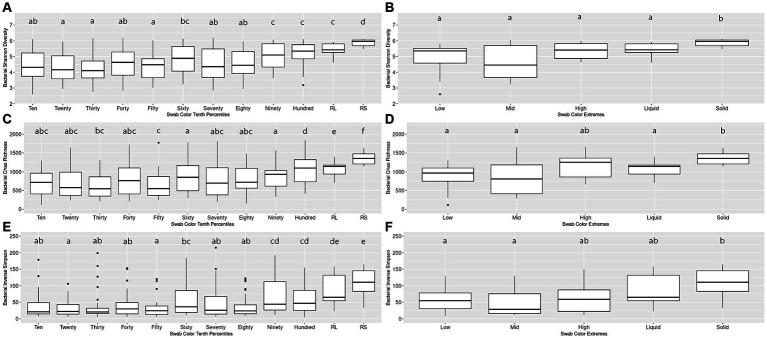
Alpha diversity boxplots for Percentiles and Extremes bacterial communities. For the bacterial Percentiles **(A,C,E)** and Extremes **(B,D,F)** sample sets, Shannon’s diversity **(A,B)**, Chao richness **(C,D)**, and Inverse Simpson **(E,F)** scores were calculated. Letters indicate significant pairwise comparisons.

An initial pairwise comparison of alpha diversity metrics within the Percentiles set was performed. When comparing the lightest group (Tenth Percentile) to the darkest (Hundredth Percentile), the lightest group had significantly lower Shannon’s diversity, Chao richness, and Inverse Simpson scores. The lightest group also differed significantly from the rumen liquid and rumen solid samples (Kruskal-Wallis, *p* < 0.05; Figures 2A,C,E). In contrast, the darkest group (Hundredth Percentile) did not have a significant difference in alpha diversity scores when compared to the rumen liquid group but differed from the rumen solids group (*p* < 0.05; Figures 2A,C,E). Overall, the hundredth percentile group had higher alpha diversity metrics relative to the lower groups, with alpha diversity increasing with greater darkness scores.

We then compared the alpha diversity metrics within the Extremes set and found no difference between the Low and High groups when assessed by any of the three diversity metrics (Figures 2B,D,F). The Low and High groups both did not differ from the rumen liquids, although the Low group differed from the rumen solids by all three metrics while the High group differed from the solids by only the Shannon’s diversity metric. Using Spearman’s rank correlation, we then compared darkness scores with the scores of the three alpha diversity metrics. We found a significant relationship between the darkness scores and all three metrics, with metrics increasing as the darkness scores increased (*p* < 0.05; Supplementary Figures S1A–C). Complete results of the pairwise comparisons can be found in Supplementary Table S2.

**Figure 3:**
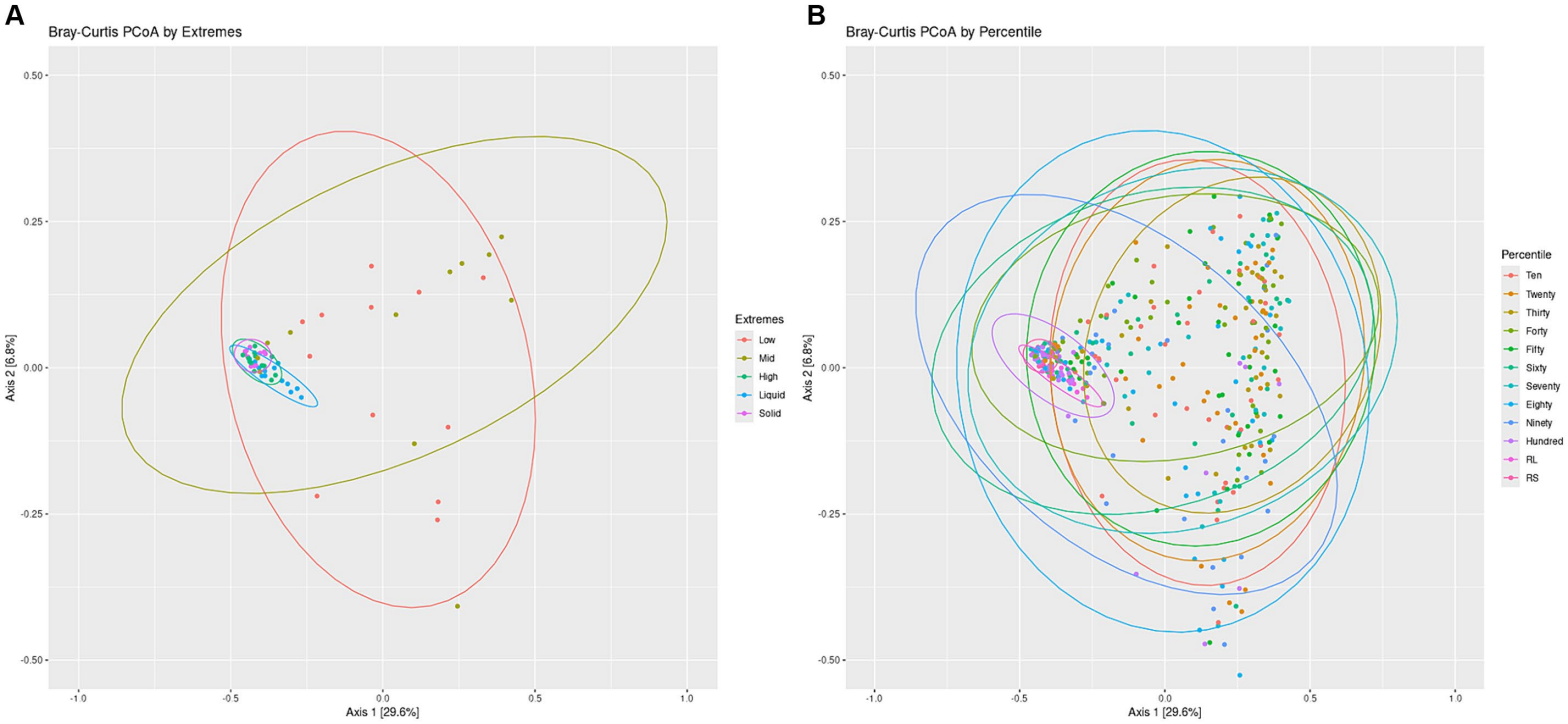
Bacterial communities Bray-Curtis PCoA by Extremes and Percentiles sets. A PCoA was generated using Bray-Curtis distances of the bacterial communities. Standard error ellipses were drawn for the Extremes **(A)** and Percentiles **(B)** sets.

To visualize the differences in overall community structure within the Percentiles and Extremes sets, Bray-Curtis dissimilarities were visualized as PCoA plots (Figure 3). In the Extremes set, we observed a larger spread for the Low group compared to the High group. The High group showed tighter clustering and overlapped much closer with the rumen liquid and solids groups than the Low group (Figure 3A).

In the Percentiles set, we observed similar results as the Extremes set. We found tight clustering of the rumen solids and rumen liquids, which overlapped with the Hundredth percentile group along with a larger spread for the groups associated with the lighter swabs (Figure 3B). Testing variance of the groups indicated a significant difference between the groups (Figure 3; *p* < 0.05) and similar results were obtained using the Jaccard index and Unweighted Unifrac metric (Supplementary Figure S2; *p* < 0.05). The structure of the communities, as represented by the top 10 phyla, showed that the lighter swab groups have a higher abundance of Proteobacteria and Actinobacteria, while the darkest swab groups look more similar to the rumen solids (Supplementary Figure S3).

To test if there was a relationship between the location of the points with the darkness score of the points, we used the X-and Y-axis locations of all swabs of the PCoA plot to perform a linear regression of the darkness scores against the X-and Y-axis points (Supplementary Figure S4A). This test showed a significant correlation between the darkness scores and the swab points on the X-axis with no correlation to the Y-axis. To further confirm if the darkness of the swab was associated with the rumen, we calculated the average distance of each swab to the rumen solids and rumen liquids. Spearman’s rank sum correlation identified a significant correlation between the darkness of the swabs and the distance of that swab’s point on the PCoA to both the rumen liquid and rumen solid groups, with the distance decreasing as the darkness score of the swab increased (Supplementary Figures S5A,B).

We then set out to determine what ASVs were driving the differences between the lightest and darkest groups within the Percentiles and Extremes sets using a SIMPER analysis and Kruskal-Wallis test. Within the Extremes set, 9 ASVs between the Low and High groups were identified including 2 ASVs (ASV3 & ASV7) that had higher abundances in the High groups and were classified into the genera *Succiniclasticum* (ASV3) and *Prevotella* (ASV7). The remaining 7 ASVs were in higher abundances in the Low groups, and classified to Unclassified Pasteurellaceae, unclassified Neisseriaceae, *Rothia*, *Streptococcus*, *Bibersteinia*, and *Moraxella* (Supplementary Figure S6).

Within the Percentiles group, we found 13 ASVs as being significantly different, including 3 ASVs (ASV1: *Succinivibrionaceae UCG-001*, ASV3, and ASV7) with higher abundances in the Hundredth percentile group and 10 with higher abundances in the Tenth percentile group. Six of those 10 were also identified in the previous seven ASVs from the Extremes set comparison with the remaining four classified to *Bibersteinia*, *Streptococcus*, and *Alysiella*. Complete tables of all pairwise SIMPER/Kruskal-Wallis comparisons can be found in Supplementary Table S3.

We also identified 471 bacterial ASVs meeting the criteria for being considered “highly prevalent” (>80% prevalence across samples) in either the rumen liquid or solid fractions (Liquid: 242 Total, 75 Unique; Solid: 396 Total, 229 Unique). Our correlation tests indicated a significant relationship between darkness scores of the samples and the percentage of highly prevalent rumen solid or liquid ASVs found in the swabs (Supplementary Figures S7A,B).

### 3.4 Fungi

#### 3.4.1 Sequencing and cleanup

Fungal sequencing resulted in a total of 18,038,649 reads, with an average of 44,104 reads per sample (±28,174) and ranging from 9,997–462,690 reads. After filtering, 4,943,507 reads remained, with an average of 12,087 reads per sample (±11,970). The number of reads per sample ranged from 2,120 to 196,930. After cleanup and normalization, 395 samples remained: 376 swabs, 7 rumen liquid, and 12 rumen solids.

#### 3.4.2 Correlations between the darkness of the swabs and the fungal community

**Figure 4:**
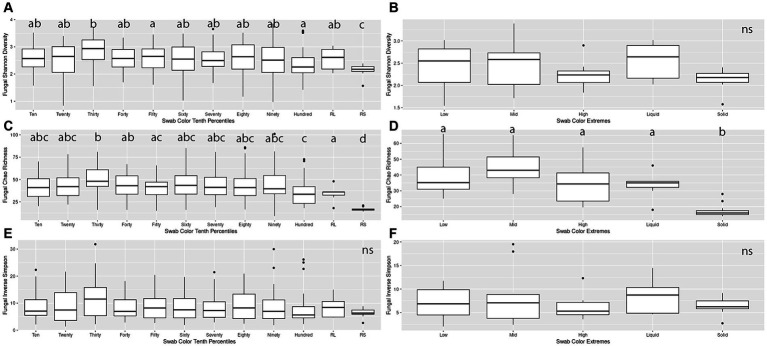
Alpha diversity boxplots for Percentiles and Extremes fungal communities. For the fungal Percentiles **(A,C,E)** and Extremes **(B,D,F)** sample sets, Shannon’s diversity **(A,B)**, Chao richness **(C,D)**, and Inverse Simpson **(E,F)** scores were calculated. Letters indicate significant comparisons.

When making pairwise comparisons of alpha diversity metrics within both the Percentiles and Extremes sets, we saw fewer differences in alpha diversity between fungal communities than in the bacterial microbiota. In the Percentiles set, the rumen solid group was the only group that was consistently different from the other groups, with overall lower scores. These findings were similar when assessed using Chao’s richness, although no significant differences were found when using Inverse Simpson’s (Figures 4A,C,E).

When comparing groups in the Extremes set, we found similar results as the Percentiles set. There were no significant differences between any of the groups when measured by Shannon’s and Inverse Simpson’s. When measured by Chao’s Richness, the rumen solids group was significantly different from all the groups (Figures 4B,D,F). A correlation analysis of the darkness scores against the three alpha diversity metrics indicated a significant relationship between the increase in darkness scores and a decrease in Shannon diversity and Chao richness (Spearman’s rank correlation; *p* < 0.05; Supplementary Figures S1D–F).

**Figure 5:**
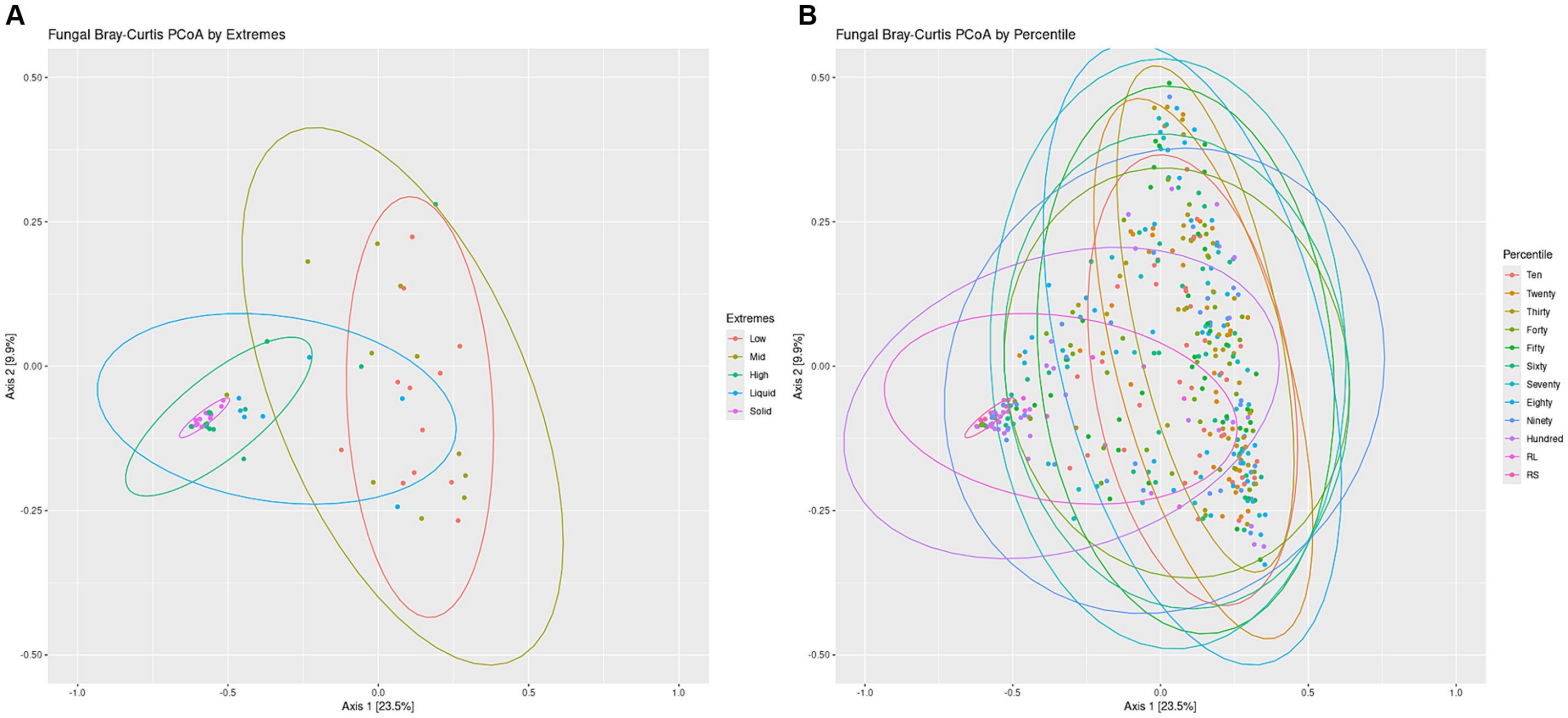
Fungal communities Bray-Curtis PCoA by Extremes and Percentiles sets. A PCoA was generated using Bray-Curtis distances of the fungal communities. Standard error ellipses were drawn for the Extremes **(A)** and Percentiles **(B)** sets.

Overall structures of the communities were visualized by generating a bar chart of the top 10 phyla within the sets. For both the Percentiles and Extremes sets, Neocallimastigomycota was found to dominate the rumen solids whereas rumen liquids had a higher abundance of Ascomycota and Basidiomycota (Supplementary Figures S3C,D). In both sets, the groups associated with the darker swabs were more similar to the rumen samples. Visualizing beta-diversity via PCoA of Bray-Curtis distances revealed a large spread between Low and Mid groups in the Extremes set, similar to the rumen liquids. The High darkness group overlapped with both rumen groups and had a smaller spread compared to the other swab groups (Figure 5A). For the Percentiles set, a large spread was observed throughout the percentile groups, including the Hundredth group, which was centered more closely over the rumen solids and liquids group (Figure 5B).

We then performed a linear regression of the darkness scores to the position of the points on the PCoA axes and found a significant relationship between swab darkness scores and their X-axis values (Supplementary Figure S4B; *p* < −0.05). There was no significant relationship between darkness scores and the Y-axis values. There was also a significant correlation between the darkness scores and the average distance of the points from the rumen solids samples, but no significant correlation against the rumen liquid samples (Supplementary Figures S5C,D).

A SIMPER and Kruskal-Wallis analysis of the Percentile groups identified 13 ASVs as driving differences between the Tenth and Hundredth Percentile groups. Of these, seven were in higher abundances in the Hundredth Percentile group, as well as in the rumen solids and liquids groups (ASV2: *Piromyces*, ASV3: Unclassified, ASV4: *Neocallimastix*, ASV5: *Caecomyces*, ASV14: *Neocallimastix*, ASV19: *Caecomyces*, ASV32: Unclassified Neocallismatigaceae). The remaining six were in higher abundances in the Tenth Percentile group (ASV1: *Debaryomyces*, ASV6: Unclassified, ASV18: Unclassified Ascomycota, ASV21: *Alternaria*, ASV43: *Diutina*, ASV54: Unclassified) (Supplementary Figure S8). Within the Extremes set, eight ASVs were identified, with six being in higher abundances in the High group and rumen groups (ASV2, ASV3, ASV4, ASV5, ASV19, and ASV32). The two that were in higher abundances in the Light group were ASV1 and ASV8 (*Agaricus silvicolae-similis*) (Supplementary Figure S8).

We identified 32 fungal ASVs highly prevalent in either the rumen liquid or solid fractions (Liquid: 26 Total, 21 Unique; Solid: 11 Total, 6 Unique). We also found a significant relationship between darkness scores of the samples and the percentage of highly prevalent rumen solid and liquid ASVs found in the swabs (Supplementary Figures S7C,D).

## 4 Discussion

The application of microbiome science to the agriculture industry requires overcoming a number of barriers including the ability to obtain samples in a non-invasive manner while ensuring sample integrity. Here, we further extend the utility of oral swabbing as a proxy method for sampling the ruminal microbiome of dairy cows by considering the overall quality of the swab and demonstrating the effectiveness of the oral swab in capturing the ruminal fungal community. Deployment of oral swabs on commercial dairy farms will likely find success if producers can collect convenience samples that do not interfere with general management practices.

Here, we sought to determine if sample color influences the ability of an oral swab to recapitulate the ruminal microbiome as swab color is thought to be a consequence of numerous factors such as time of sampling and personnel sampling ability. If a relationship between sample color and ruminal microbiome representation exists, oral swab color could act as an indicator of the quality of the swab at the time of sampling. We employed a novel colorimetric method for quantifying swab color and showed that the darkness of the swab is indicative of significantly different microbial communities, with darker swabs having bacterial and fungal communities that are more similar to the rumen solid and liquid communities. Future work can also include the development of other quantification methods, including using a spectrophotometer on the buffer the swab is stored in to measure darkness.

Three lines of evidence support our findings regarding the bacterial communities: (i) darker swabs had higher and more similar alpha diversity scores to ruminal solids and liquids, relative to lighter swabs (Figure 2); (ii) darker swabs exhibited a tighter range of scores, indicating a higher diversity with less variance; and (iii) a comparison of the microbial structure of the darker swabs via PCoA showed significant overlap with ruminal solids and liquids (Figure 3). Importantly, our regression analysis revealed a significant correlation between the X-axis of the PCoA and darkness scores (Supplementary Figure S4), indicating that highly prevalent ruminal bacteria are captured by darker swabs and that darkness score is a variable that influences the ability of a swab to capture the ruminal microbiome.

Given these observations, we posited that lighter swabs are less likely to recapitulate the ruminal bacterial microbiome, relative to darker swabs, due to an increased presence of oral bacteria. Indeed, our SIMPER/Kruskal-Wallis analysis identified numerous ASVs that drove the observed differences between these groups (Supplementary Figure S6). For example, three ASVs were more abundant in both the dark swabs and the rumen samples and belong to known rumen microbes in the *Succinivibrionaceae UCG-001* (ASV1), *Succiniclasticum* (ASV3), and *Prevotella* (ASV7). In contrast, we found 11 *bona fide* oral-associated ASVs in higher abundances in lighter swabs belonging to the Pasteurellaceae, Neisseriaceae, *Rothia*, *Streptococcus*, *Bibersteinia*, *Alsyiella,* and *Moraxella* (Young et al., 2020; Borsanelli et al., 2018). These results coincide with our previous work that identified these phyla and genera as oral-associated microbes (Young et al., 2020). It is important to note that dark swabs are more similar to the rumen solid fraction than the rumen liquid fraction, confirming previous findings (Young et al., 2020), and we hypothesize that darker swabs likely captured more rumen solids present in the oral cavity at the time of collection. This is likely a consequence of rumen solids spending more time in the oral cavity during rumination (i.e., chewing the cud), relative to the liquid fraction, which is quickly swallowed during rumination, leaving a higher proportion of rumen solids available for swab collection (Beauchemin, 2018).

The ability of oral swabs to capture the ruminal bacterial community is well documented, but much less is known about the other microbial members of the rumen. In short, this study presents the first analysis of oral swabs as a proxy for recapitulating the ruminal fungal community, which is known to play an important role in both plant biomass deconstruction and nutrient provisioning through VFA production (Nagaraja, 2016; Mizrahi, 2012; Cammack et al., 2018). Our findings support the hypothesis that oral swabs are a good proxy for characterizing the ruminal fungal community and that, similar to the bacterial microbiome, is correlated to swab color. Our initial analysis of alpha diversity across swab color groups revealed that, although they did not differ from rumen liquids, darker swabs differed significantly from lighter swabs when compared against ruminal solids (Figure 4). Importantly, darker swabs decreased significantly in alpha diversity, relative to lighter swabs, and approached values similar to the ruminal solid fungal microbiome. This finding is in line with our hypothesis that darker swabs are better at capturing ruminal solids and further mirrors what is known about the ruminal fungal microbiome, as ruminal solids exhibit lower overall richness and diversity, relative to ruminal liquids.

Further lines of evidence are revealed through our PCoA analysis of the fungal communities, which showed that darker swabs overlapped more with rumen solids and liquids and with a tighter spread when compared to lighter swabs (Figure 5). Regression of swab darkness scores against our PCoA X-axis values indicated a significant relationship between the darkness of the swab and its position on the PCoA plot (Supplementary Figure S4B), with swabs increasing in similarity with ruminal solids as they became darker. In contrast, there was a lack of significance between darker swabs and the rumen liquids, suggesting a wide variability in the structure of the rumen liquid communities. As such, oral swabs may not be as effective at recapitulating the ruminal liquid fungal microbiome, and comparison of the ruminal liquid fungal ASVs captured by the swabs indicated that lighter swabs contained more highly prevalent ruminal liquid fungi ASVs (*p* < 0.0498, Supplementary Figure S7D). This may be due to the abundant ruminal solid fungi “washing out” the ruminal liquid members from darker swabs.

Finally, we sought to determine if darker swabs are more capable of capturing ruminal fungi, relative to lighter swabs. We found a number of ASVs that drove the observed differences between color groups (Supplementary Figure S8). Darker swabs were dominated by seven ASVs that classified to the phylum Neocallimastigomycota. Members of this phylum are well-known taxa within the ruminal solid community as they play key roles in plant fiber degradation (Cox et al., 2021; Gruninger et al., 2014). In contrast, seven ASVs including those belonging to the *Debaryomyces*, *Agaricus silvicolae-similis*, and *Diutina catenulate*, were found to dominate lighter swabs and are known to be associated with the oral and nasal microbiomes of cattle, dairy cow feed, and agricultural environments (e.g., soils, water, etc) (O’Brien et al., 2018; Kumar et al., 2015; Centeno-Martinez et al., 2023; Rosenbaum et al., 2019).

Our study showed that, on average, ~50% of the core ruminal community was captured by our swabs (Supplementary Figure S7). We also found that above a darkness score of 0.5, there was much less variability in the percentage of ASVs captured, with most swabs capturing upwards of 70% of the highly prevalent ruminal ASVs in the bacterial community, confirming our previous report (Young et al., 2020). This was less pronounced in the fungal community, with darkness scores negatively correlated with the percent of rumen liquid ASVs captured (Supplementary Figure S7). Further research is required to determine what percentage would be sufficient to characterize major differences between animals with distinctly different production metrics, as previous studies have noted significantly different microbial communities between animals of differing milk productions (Jewell et al., 2015; Jami et al., 2014). We hypothesize that darker swabs are likely a result of time since rumination, with swabs collected closer to active rumination resulting in darker swabs. Further work would be required to confirm this. Until then, researchers can attempt to maximize the darkness of the swab by sampling during or as close to rumination as possible. Real-time monitoring devices, such as rumination collars, can be employed to aid in this timing.

We have previously tested using abundances in swabs to predict abundances in the rumen. This would allow for a more thorough comparison of the rumen community between animals. Our previous attempts were not very successful (Young et al., 2020). Swab color may thus be an important factor in the success of the regression model that could be included in future attempts. In addition, the development of an objective method to filter environmental and oral-associated bacteria and fungi from oral swabs could produce a dataset with microbiomes that are more representative of their respective rumen populations, regardless of darkness score, so long as sufficient sequencing depth has been attained to capture the entire community, further increasing the chance of success for a regression model. Successful regression of the ruminal microbial community from swab data would allow for greater flexibility in the darkness of swab required to characterize the rumen bacterial community, assuming our hypothesis described earlier regarding community structure holds true.

In sum, our results demonstrate that swab color is an indicator of its ability to capture the ruminal microbiome. We showed that darker swabs are more effective at replicating both the ruminal bacterial community and the ruminal solid fungal community. Although lighter swabs were not as representative of the ruminal microbiome as compared to darker swabs, they did exhibit somewhat similar community structure to the rumen, as shown in our PCoAs. Future work should explore the potential for a pre-enrichment prior to DNA extraction so as to increase the abundance of rumen-originated DNA, thereby allowing lighter swabs to be more representative of the ruminal community as darker swabs.

Finally, our results suggest that one of the main drivers of the differences observed between light and dark swabs is the level of variability present between the groups. Darker swabs have less variability in their communities compared to lighter swabs. We hypothesize that the overall structure of the rumen microbial community is maintained in lighter swabs but is proportionally smaller to darker swabs as oral and environmental bacteria represent a larger proportion of the collected DNA. One caveat to this is the collection of recently chewed feed will also make the swab darker, and we currently cannot differentiate between swabs darkened by rumen contents or feed.

## 5 Conclusion

Here, we demonstrate that oral swabs are an effective proxy for capturing both the ruminal bacterial and fungal microbiomes. Moreover, we show that oral swab color is indicative of the ability of a swab to recapitulate the ruminal microbiome and developed a novel colorimetric method to quantify swab color. Importantly, we found that darker swabs are more strongly correlated to the ruminal microbiome than lighter swabs, and swabs with darkness scores >0.5 were capable of consistently capturing upwards of 70% of the most highly prevalent bacteria in the rumen. Our study also demonstrates for the first time that oral swabbing is effective in capturing the ruminal fungi, which will be of benefit to researchers and producers interested in understanding the complete ruminal microbiome. Based on these results, we suggest that oral swabbing is an effective approach for capturing the ruminal microbiome and that the variance in sample quality likely to be encountered when deployed on commercial farms via convenience sample can be effectively accounted.

## Data Availability

The dataset generated and analyzed during the current study are available in the NCBI’s Short Read Archive under BioProject number PRJNA1117920. Python and R code is available at https://github.com/JSkar/SwabColor.

## Supplementary material

SUPPLEMENTARY Table S1 Primer sequences used for 16S and ITS sequencing.

SUPPLEMENTARY Table S2 Complete pairwise alpha diversity comparisons.

SUPPLEMENTARY Table S3 All ASVs with >1% SIMPER results with Kruskal-Wallis results.
