# Corrigendum: Oral swabs as a proxy for direct ruminal microbiome sampling in Holstein dairy cows is correlated with sample color

**DOI:** 10.3389/fmicb.2024.1501270

**Published:** 2024-11-13

**Authors:** Joseph H. Skarlupka, Madison S. Cox, Andrew J. Steinberger, Dino L. Sbardellati, Jennifer C. McClure, Derek M. Bickhart, Andrew J. Scheftgen, Ibrahim Zuniga-Chaves, Luke A. Wolfe, Eric Paget, Charles Skadron, Nithya Attipetty, Garret Suen

**Affiliations:** ^1^Microbiology Doctoral Training Program, University of Wisconsin–Madison, Madison, WI, United States; ^2^Department of Bacteriology, University of Wisconsin–Madison, Madison, WI, United States; ^3^Department of Allergy and Infectious Disease, University of Washington School of Medicine, Seattle, WA, United States; ^4^Microbiology Graduate Group, University of California, Davis, Davis, CA, United States; ^5^USDA Dairy Forage Research Center, Madison, WI, United States; ^6^Hendrix Genetics, Boxmeer, Netherlands

**Keywords:** buccal swab, oral community, rumen microbiota, swab color, bacteria, fungi

In the published article, there was a mistake in a reference made in the Discussion. The statement “We also found that above a darkness score of 0.5, there was much less variability in the percentage of ASVs captured, with most swabs capturing upwards of 70% of the highly prevalent ruminal ASVs in the bacterial community, confirming our previous report (Edwards et al., 2023).” was incorrectly referenced.

The correct reference for the statement is “We also found that above a darkness score of 0.5, there was much less variability in the percentage of ASVs captured, with most swabs capturing upwards of 70% of the highly prevalent ruminal ASVs in the bacterial community, confirming our previous report (Young et al., 2020).”

The authors apologize for this error and state that this does not change the scientific conclusions of the article in any way. The original article has been updated.

