# Supplementary material for: Oral swabs as a proxy for direct ruminal microbiome sampling in Holstein dairy cows is correlated with sample color
Source: Front Microbiol. 2024 Sep 17;15:1466375. doi: 10.3389/fmicb.2024.1466375 (PMC11443345; doi:10.3389/fmicb.2024.1466375)
Supplement: Supplementary file 7 [file Data_Sheet_4.PDF]

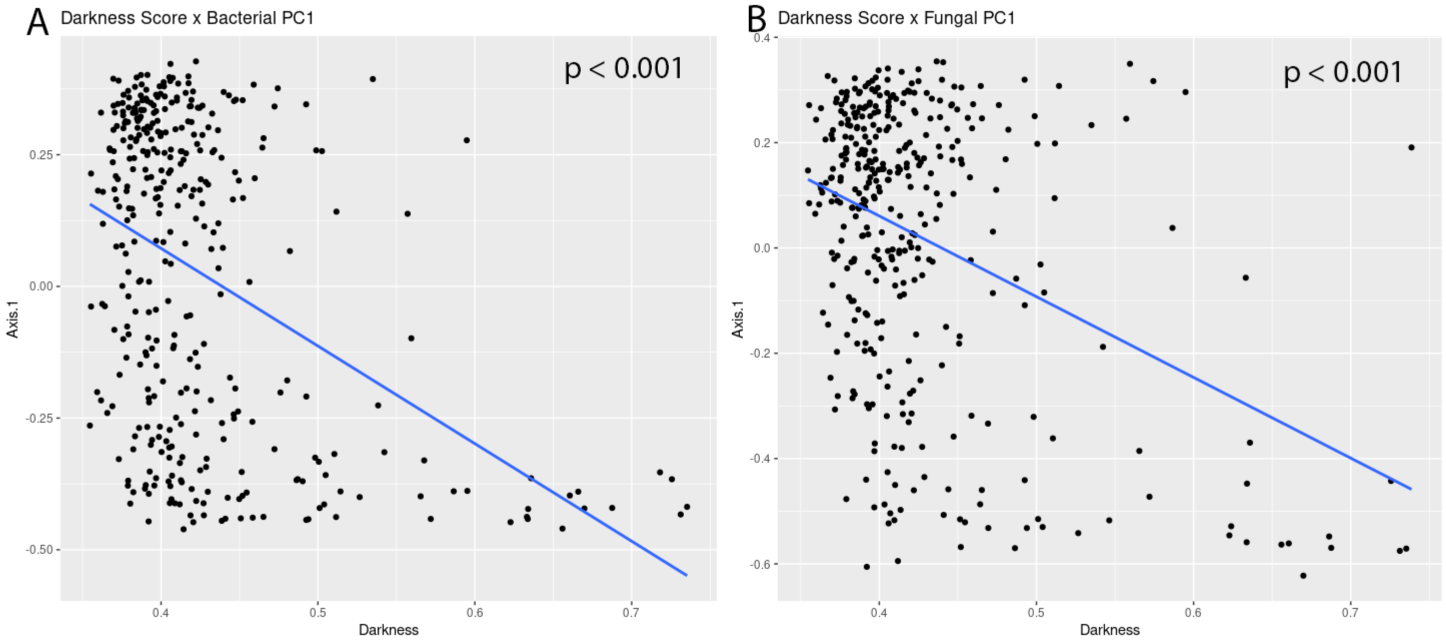

Supplementary Figure 4. Spearman's rank correlation of darkness scores with bacterial and fungal PCoA axis values.
